# Genome-wide identification and expression analysis of TCP family genes in *Catharanthus roseus*

**DOI:** 10.3389/fpls.2023.1161534

**Published:** 2023-04-12

**Authors:** Juan Hao, Lijun Zheng, Yidie Han, Hongshan Zhang, Kailin Hou, Xueshuang Liang, Cheng Chen, Zhijing Wang, Jiayi Qian, Zhihao Lin, Zitong Wang, Houqing Zeng, Chenjia Shen

**Affiliations:** ^1^ College of Life and Environmental Sciences, Hangzhou Normal University, Hangzhou, China; ^2^ Zhejiang Provincial Key Laboratory for Genetic Improvement and Quality Control of Medicinal Plants, Hangzhou Normal University, Hangzhou, China; ^3^ Kharkiv Institute, Hangzhou Normal University, Hangzhou, China; ^4^ College of Pharmacy, Hangzhou Normal University, Hangzhou, China

**Keywords:** cis-element, elicitor, expression pattern, stress, TCP family, qRT-PCR, terpenoid indole alkaloids

## Abstract

**Introduction:**

The anti-tumor vindoline and catharanthine alkaloids are naturally existed in Catharanthus roseus (C. roseus), an ornamental plant in many tropical countries. Plant-specific TEOSINTE BRANCHED1/CYCLOIDEA/PCF (TCP) transcription factors play important roles in various plant developmental processes. However, the roles of C. roseus TCPs (CrTCPs) in terpenoid indole alkaloid (TIA) biosynthesis are largely unknown.

**Methods:**

Here, a total of 15 CrTCP genes were identified in the newly updated C. roseus genome and were grouped into three major classes (P-type, C-type and CYC/TB1).

**Results:**

Gene structure and protein motif analyses showed that CrTCPs have diverse intron-exon patterns and protein motif distributions. A number of stress responsive cis-elements were identified in promoter regions of CrTCPs. Expression analysis showed that three CrTCP genes (CrTCP2, CrTCP4, and CrTCP7) were expressed specifically in leaves and four CrTCP genes (CrTCP13, CrTCP8, CrTCP6, and CrTCP10) were expressed specifically in flowers. HPLC analysis showed that the contents of three classic TIAs, vindoline, catharanthine and ajmalicine, were significantly increased by ultraviolet-B (UV-B) and methyl jasmonate (MeJA) in leaves. By analyzing the expression patterns under UV-B radiation and MeJA application with qRT-PCR, a number of CrTCP and TIA biosynthesis-related genes were identified to be responsive to UV-B and MeJA treatments. Interestingly, two TCP binding elements (GGNCCCAC and GTGGNCCC) were identified in several TIA biosynthesis-related genes, suggesting that they were potential target genes of CrTCPs.

**Discussion:**

These results suggest that CrTCPs are involved in the regulation of the biosynthesis of TIAs, and provide a basis for further functional identification of CrTCPs.

## 1 Introduction

The TEOSINTE BRANCHED1/CYCLOIDEA/PCF (TCP) family belongs to a type of plant-specific transcription factors (TFs) that played roles in diverse aspects of growth and developmental processes (Wang et al., 2022). The TCP family was named after its firstly discovered genes in maize (TEOSINTE BRANCHED1), *Antirrhinum* (CYCLOIDEA), and rice (PCF) (Doebley et al., 1997; Kosugi and Ohashi, 1997; Luo et al., 1999). Based on TCP domain, TCPs were grouped into TCP-P type (mainly PCF class) and C-type type consisting of the CIN class and the CYC/TB1 class (Navaud et al., 2007). The classic TCP domain contains a noncanonical basic helix–loop–helix motif, which is defined by structural feature and is unrelated to the DNA binding bHLH domain (Xu et al., 2014).

Increasing evidences suggested that *TCP* genes played roles in a wide range of growth- and development-related processes (Hao et al., 2012). In *Arabidopsis*, several *TCP* genes were found to be involved in microRNA-guided controlling leaf development (Palatnik et al., 2003). AtTCP2, AtTCP3, AtTCP11, and AtTCP15 were identified to bind to different components of the core circadian clock, such as LATE ELONGATED HYPOCOTYL1 and PSEUDO RESPONSE REGULATOR5 (Giraud et al., 2010). AtTCP20 acts as an activator of nitrate assimilatory gene, *NIA1*, and an inhibitor of the G2/M cell-cycle-related gene, *CYCB1;1*, suggesting an important role of TCPs in the linking of nitrate assimilation and cell-cycle (Guan et al., 2017). AtBRANCHED1 functions as a regulator of branching signals in bud outgrowth and development by interacting with FLOWERING LOCUS T (Niwa et al., 2013). In the TCP class II, one inhibitory gene (*BRANCHED-like*) and two activating genes (*JAW-like* and *TCP5-like*) are involved in axillary branch outgrowth (van Es et al., 2019). Interestingly, TCP family was considered to be involved in hormone signaling pathways. A TCP class I member, AtTCP8, modulates the expression of several brassinosteroid-responsive genes, such as *BRASSINAZOLE-RESISTANT1* and *BRASSINAZOLE-RESISTANT2* (Spears et al., 2022). A cotton TCP, GrTCP11, inhibits root hair elongation by reducing JA signaling pathway in heterologous expressed *Arabidopsis* system (Hao et al., 2021).

Recent studies revealed that TCP family played essential roles in secondary metabolism. In *Ginkgo biloba*, three TCP family members, such as *GbTCP03*, *GbTCP04*, and *GbTCP07*, were reportedly involved in flavonoid biosynthesis (Yu et al., 2022b). Heterologous overexpression of *GbTCP5* gene in *Arabidopsis* increased the lignin biosynthesis by binding to TCP II type *cis*-acting elements (Wang et al., 2020). *Marchantia polymorpha TCP1*, a TCP-P type gene, enhanced the formation of secondary metabolites, such as aminochromes (Busch et al., 2019). In tea plant, CIN-type *CsTCP3/4* and PCF-type *CsTCP14* modulated the transactivation activity of anthocyanin synthase and anthocyanidin reductase by forming a CsTCP3-CsTT8 heterodimer (Yu et al., 2021).

*Catharanthus roseus* (L.) G. Don, a diploid plant native to Madagascar, is grown as an ornamental plant in many tropical countries (Nejat et al., 2015). *C. roseus* extracts have historically been applied to treat a wide range of diseases like diabetes, astringent, diuretic and cough remedy for many years (Nammi et al., 2003). Researchers identified the occurrence of vinblastine and vincristine in *C. roseus*, belonging to a specific type of terpenoid indole alkaloids (TIAs) that were used in cancer therapy (Kellner et al., 2015). Due to their very low yield, a variety of artificial induction methods have been employed to enrich the *C. roseus* alkaloids, including callus suspension culture, chemical elicitation and stress inducer (Zhao et al., 2001; Rizvi et al., 2016). It is interesting that elicitors enhanced the content of key alkaloids in *C. roseus* by altering the expression of TIA biosynthesis-related genes (Soltani et al., 2022). Several common elicitors, such as methyl jasmonate (MeJA), salicylic acid (SA), ultraviolet-B (UV-B), and other environmental stresses, have been applied in the accumulation of *C. roseus* active ingredients (Ramani and Chelliah, 2007; Goklany et al., 2013; Soltani et al., 2022). However, roles of CrTCP family in the regulation of TIA biosynthesis in *C. roseus* are largely unknown (Hao et al., 2021).

To date, a large number of *TCP* genes, such as 24 TCP members in *Arabidopsis*, 35 TCP members in *Melastoma candidum*, 14 TCP members in *Cymbidium goeringii*, 30 TCP members in *Solanum lycopersicum*, 33 TCP members in *Populus euphratica*, and 21 TCP members in rice, were identified (Navaud et al., 2007; Hao et al., 2012; Parapunova et al., 2014; Ma et al., 2016; Liu et al., 2019; Li et al., 2022). However, *TCP* gene family in *C. roseus* genome has not been identified, and its roles in development and metabolism are largely unknown. In this study, a detailed bioinformatic analysis of *TCP* family genes based on the newly published *C. roseus* genome and their expression patterns in different tissues and in responses to elicitors were carried out. Our research will help to understand the potential roles of CrTCPs in active ingredient biosynthesis and provide valuable information for the molecular breeding of *C. roseus*.

## 2 Materials and methods

### 2.1 Plant materials and growth condition

*Catharanthus roseus* seedlings were planted in a greenhouse in Hangzhou Normal University, Hangzhou, China, at a temperature of 25 ± 2°C with a light/dark cycle of 8/16 h and 65%–75% relative humidity. Forty-five-day-old seedlings in pots were used for the abiotic stress treatments.

### 2.2 Abiotic stress treatments

For UV-B treatments, plants were randomly separated into different groups with three pots each. One group was kept in standard growth condition and used as control. Three other groups were treated with UV-B radiation for 3 h, 6 h, and 24 h, respectively. Artificial UV-B at 10 μmol m^−2^s ^−1^ was made by a fluorescent lamp (Q-Lab narrowband UV-B type), wrapped with a 3 mm transmission filter (Schott, Mainz, Germany), and measured by an Apogee UVB intensity meter (Yang et al., 2020). For MeJA treatments, one group of seedlings sprayed with ddH_2_O was used as control. MeJA at a concentration of 100 μM was sprayed onto the leaves of *C. roseus* seedlings for 1 h, 3 h, and 6 h, respectively (Zhan et al., 2018). For each treatment, three independent repetitions were applied.

### 2.3 Identification of CrTCP family genes

The protein sequences of TCPs in *C. roseus* were searched from the published genome (Kellner et al., 2015). The local BLATX program was used to identify potential CrTCP proteins using the ‘cro_v2.proteins.fasta’ file. Using the resulting protein IDs, the full-length CrTCP family gene sequences were extracted from the ‘cro_v2.transcripts.fasta’ file. All the potential CrTCP protein sequences were analyzed using InterPro program to check their domain features.

### 2.4 Phylogenetic tree, gene structure and protein domain analysis

The sequences of CrTCP proteins were used for phylogenetic analysis. A multiple sequence alignment was carried out using the full length sequences of CrTCP proteins by ClustalW software. The phylogenetic tree was constructed using CrTCP protein sequences by MEGA 6.1 with neighbor-joining method. The exon-intron pattern of CrTCP genes was identified using GSDS 2.0 online tool (http://gsds.gao-lab.org/). Bootstrap values of 1000 was applied to phylogeny test and *P* distance of protein sequence was used to analyze substitution rates. Pfam program in InterPro database (https://www.ebi.ac.uk/interpro/entry/pfam/#table) were used to identify motifs present in the CrTCP proteins. ‘PSORT WWW Server’ (https://psort.hgc.jp/) tool was used to predict the subcellular localization of CrTCP proteins.

### 2.5 *Cis*-element analysis

The 2000 bp promoter sequences of *CrTCP* genes were extracted from the *C. roseus* genome using ‘cro_v2_asm.fasta’ file. The promoters were uploaded and scanned using the PlantCARE program.

### 2.6 Measurement of terpenoid indole alkaloid contents

Collected leaf samples were dried at 60°C for 24 h and grinded in a mortar. The extraction of TIAs from *C. roseus* leaves was performed using methanol ultrasonic extraction method. *C. roseus* leaves were freeze dried and screened through 40 mesh sieve. About 100 mg dry samples were measured. After added with 1.5 mL of extraction agent, samples were ultrasonically extracted at 37 °C for 30 min. The supernatants were obtained by centrifugation at 12000 rpm for 10 min. The content of TIAs is determined based on the previous published HPLC method (Zhu et al., 2015). High performance liquid chromatographic conditions: the liquid to be tested is tested by HPLC (Waters e2695) instrument, and the chromatographic column is Waters C18 (4.6 mm × 250 mm); mobile phase: A: 0.025 M ammonium acetate solution; B: acetonitrile; gradient elution procedure: 0~5 min, 70% - 60% A; 5~8 min, 60% -55% A; 8~22 min, 55% -50% A; 22~27 min, 50% -30% A; 27~30 min, 30%-70% A; flow rate: 1.0 mL/min; detection wavelength: 220 nm. The HPLC chromatograms were showed in **Supplemental File 1**.

### 2.7 RNA isolation and quantitative real-time PCR

RNAs were isolated from each sample group using a plant TRIzol reagent (Invitrogen, Shanghai, China). RNA quantity and quality were analyzed using a NanoDrop ND-430 spectrophotometer (Agilent, Santa Clara, CA, USA). About 1.0 μg RNAs of each sample were reverse transcribed to cDNA library using ReverAid First Strand cDNA Synthesis Kit (Thermo Scientific, Shanghai, China). qRT-PCR assay (three independent repeats) was applied using a SYBR Premix Ex Taq Kit (TaKaRa, Dalian, China) on an ABI PRISM 7700 DNA Sequence Detection System (Applied Biosystems, Shanghai, China). All primers were listed in **Table S1**.

### 2.8 Statistical analysis

Significant difference in TIA contents between two treatment groups was analyzed by a one-way analysis of variance method with a student’s test at a *P* value < 0.01. Gene expression and chemical contents were analyzed using three independent biological repeats.

## 3 Results

### 3.1 Identification of TCP family members in *C. roseus*

Fifteen potential *CrTCP* family genes were screened and identified in *C. roseus* based on its newly uploaded genome dataset. The open reading frame (ORF) sequence of the *CrTCP* genes range from 438 bp (*CrTCP12*) to 1878 bp (*CrTCP13*), the molecular weight of the proteins range from 17.2 kDa to 68.6 kDa, and the corresponding isoelectric points vary from 10.48 to 6.01. The grand average of CrTCP hydrophilic values is negative, suggesting that they are strong hydrophilic proteins. Subcellular location of all CrTCP proteins was predicted to be in the nucleus, suggesting that they function as TFs in the nucleus (**Table 1**).

**Table 1:** The basic information of all identified TCP family members in *C. roseus*.

Gene name	Gene ID	Location	ORF (bp)	Length (aa)	MW (Da)	PI
CrTCP1	CRO_T110123	scaffold_156:22232-23767	1533	511	54292.9	8.27
CrTCP2	CRO_T102976	scaffold_104:816800-818368	1071	357	40095.3	8.22
CrTCP3	CRO_T112581	scaffold_175:138760-143950	1449	483	51985.4	9.21
CrTCP4	CRO_T131197	scaffold_54:1786611-1787879	1266	422	44773.1	7.41
CrTCP5	CRO_T133475	scaffold_62:2166697-2168590	1848	616	67731.5	9.08
CrTCP6	CRO_T104052	scaffold_11:4828739-4829746	1005	335	37108.7	6.3
CrTCP7	CRO_T133000	scaffold_60:714383-721918	1422	474	53940.8	9.48
CrTCP8	CRO_T101037	scaffold_1:93042-93734	783	261	29771.5	9.47
CrTCP9	CRO_T133640	scaffold_63:1997535-1998338	801	267	27450.6	9.51
CrTCP10	CRO_T126653	scaffold_401:8966-9859	891	297	31970.1	9.07
CrTCP11	CRO_T134142	scaffold_66:805164-806918	1020	340	36220.1	9.21
CrTCP12	CRO_T119545	scaffold_256:92824-94995	438	146	17162.2	10.48
CrTCP13	CRO_T132908	scaffold_60:40536-41558	1878	626	68573.9	6.01
CrTCP14	CRO_T105570	scaffold_12:5327783-5329144	1359	453	47874.3	7.18
CrTCP15	CRO_T128670	scaffold_46:2328133-2329992	1155	385	41371.6	7.87

### 3.2 Phylogenetic and amino acid sequence analysis

**Figure 1:**
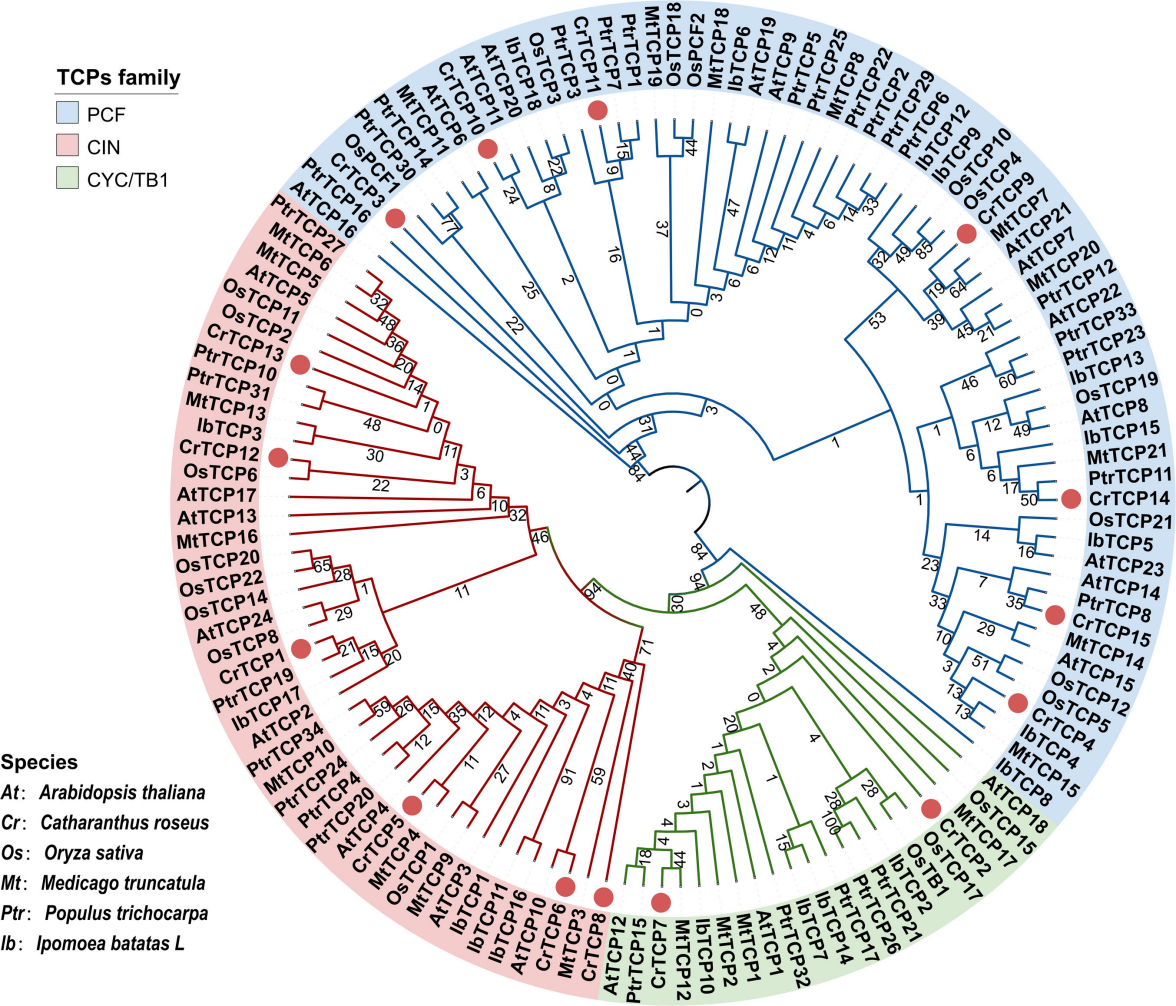
Phylogenetic analysis of CrTCP family members. Different colors of the outer ring denote the three main TCP groups. The blue arc represents PCF-type genes, the red arc represents CIN type, and the yellow arc represents CYC/TB1 type. ‘At’ indicated *Arabidopsis thaliana*, ‘Cr’ indicated *Catharanthus roseus*, ‘Os’ indicated *Oryza sativa*, ‘Mt’ indicated *Medicago truncatula*, ‘Ptr’ indicated *Populus trichocarpa*, and ‘Ib’ indicated *Ipomoea batatas*. Red dots indicated the CrTCPs.

TCP proteins from *C. roseus*, model plant *Arabidopsis thaliana* and *Oryza sativa*, *Medicago truncatula*, *Populus trichocarpa*, and *Ipomoea batatas* were selected for phylogenetic tree construction. The phylogenetic analysis showed that all TCP family members could be divided into three main subclasses: TCP-P type, TCP-C type, and CYC/TB1 type. Seven CrTCPs (CrTCP3, CrTCP4, CrTCP9, CrTCP10, CrTCp11, CrTCP14, and CRTCP15) were in the TCP-P type subclass (PCF), six CrTCPs (CrTCP6, CrTCP8, CrTCP5, CrTCP1, CrTCP13, and CrTCP12) were in the TCP-C type subclass (CIN), and only two CrTCPs (CrTCP2 and CrTCP7) were in the CYC/TB1 subclass (**Figure 1A**).

The alignment analysis showed that CrTCPs possessed a series of basic amino acid residues, followed by two helix and one loop motifs, indicating that the TCP domain is highly conserved in plants (**Figure S1A**). The protein-protein network between all CrTCPs was predicted using STRING tool. Among the CrTCP proteins, eight CrTCPs, including CrTCP1, CrTCP2, CrTCP3, CrTCP4, CrTCP5, CrTCP10, CrTCP14, and CrTCP15, formed a protein-protein network (**Figure S1B**).

### 3.3 Gene structure and protein domain analysis of CrTCP family members

**Figure 2:**
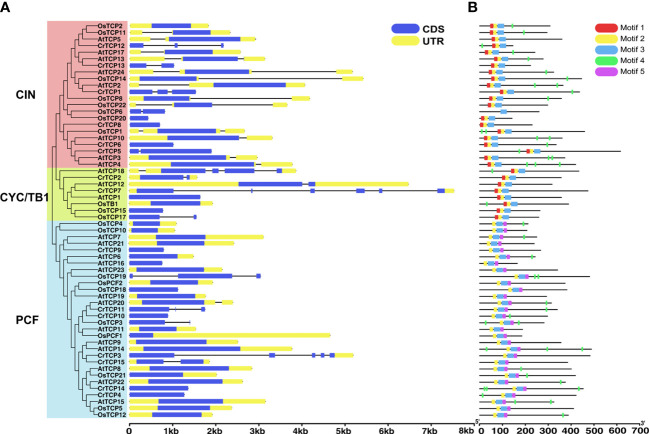
Gene structure and protein domain analysis of CrTCP family members. **(A)** Gene structure analysis of CrTCP family members. Green boxes indicate CDSs and yellow boxes indicated UTRs. All the TCPs were grouped into three classes according to their evolution relationship. **(B)** Rectangles with different colors represent different protein motifs.

Gene structure analysis showed that only four CrTCP genes, such as *CrTCP2*, *CrTCP3*, *CrTCP7*, and *CrTCP15*, contain untranslated region annotation, indicating the weak assembling quality of *C. roseus* genome. Six *CrTCP* genes, *CrTCP4*, *CrTCP8*, *CrTCP6*, *CrTCP10*, *CrTCP9*, and *CrTCP14*, contain only one exon. *CrTCP3* and *CrTCP8* have the most complex gene structures with four and five introns, respectively (**Figure 2A**). The motifs of the CrTCP proteins were analyzed by MEME program, and five motifs were set as upper bounds. The number of motifs in CrTCPs range from one to five. Most CrTCPs contain motif 2 and 3 in the *N*-terminal (**Figure 2B**). The motif 2 and 3 corresponds to the TCP domain, and other motifs correspond to highly variable region.

### 3.4 Tissue-specific expression pattern and *cis*-element analysis

**Figure 3:**
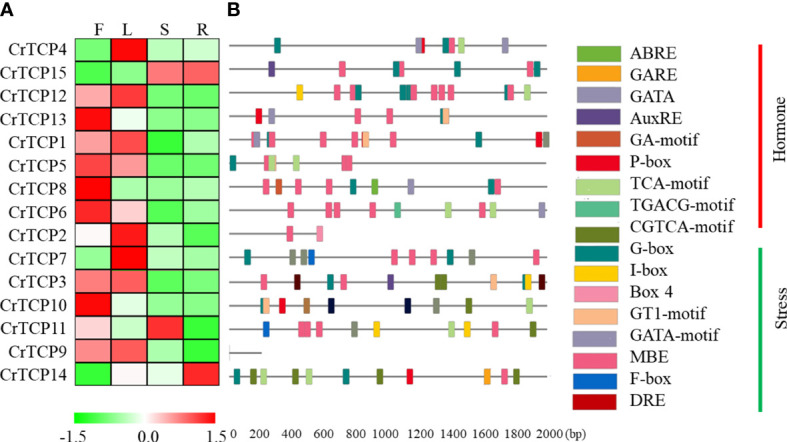
Tissue-specific expression pattern and *cis*-element analysis of CrTCP family members. **(A)** Expression analysis of CrTCP family genes in four different tissues by qRT-PCR assay. F: flowers, L: leaves, S: stem, and R: roots. Red indicated high expression level and green indicated low expression level. All the TCPs were grouped into different classes according to their evolution relationship. **(B)** Promoter analysis of CrTCP genes. Different color boxes on the black lines indicated various *cis*-elements. All cis-elements were grouped into two classes: hormone- and stress-related elements.

The tissue-specific expression of *CrTCP* gene family in different tissues, including flower, leaf, stem, and root, was analyzed by qRT-PCR. Generally, all *CrTCP* genes can be detected in at least one tissue. *CrTCP13*, *CrTCP8*, *CrTCP6*, and *CrTCP10* specifically expressed in the flowers; *CrTCP4*, *CrTCP2*, and *CrTCP7* highly expressed in the leaves; *CrTCP11* mainly expressed in the stem; and *CrTCP14* is a root specific expressed gene (**Figure 3A**).

*Cis*-element analysis showed that the promoters of *CrTCP* genes had various plant specific binding elements, including nine hormone-related elements and eight stress-related elements. The MYB binding element (MBE) were significantly enriched in the promoter regions of *CrTCP* genes. For example, *CrTCP12* (7 MBEs), *CrTCP1* (5 MBEs), and *CrTCP6* (5 MBEs) contained more than five MBEs in their promoter regions. The promoter sequences of several *CrTCP* genes contain several abiotic stress-related *cis*-elements. One GA-motif, one G-box, and two ABREs were found in the promoter region of *CrTCP8* and three W-boxes and two G-boxes were contained in the promoter region of *CrTCP7*, suggesting their potential roles in abiotic stress responses (**Figure 3B**). Due to the low quality of *C. roseus* genome, the promoter sequences of *CrTCP2* and *CrTCP9* are temporarily unavailable. Furthermore, TGACG-motif and CGTCA-motif were *cis*-elements responsive to MeJA treatment. Two TGACG-motifs and one TGACG-motif were contained in the promoter sequences of *CrTCP3* and *CrTCP6*, respectively. The CGTCA-motif was enriched in the promoter sequences of *CrTCP3* and *CrTCP14*. Our data suggested that the expression of *CrTCP3*, *CrTCP6* and *CrTCP14* might be regulated by the MeJA treatments. I-box, GT1-motif, and GATA-motif were reported to be light response elements. The promoter of *CrTCP4* contained two GATA-motifs and the promoter of *CrTCP11* contained two I-boxes, suggesting that *CrTCP4* and *CrTCP11* might be light responsive TFs.

### 3.5 Expression pattern of CrTCP family genes under various elicitors

**Figure 4:**
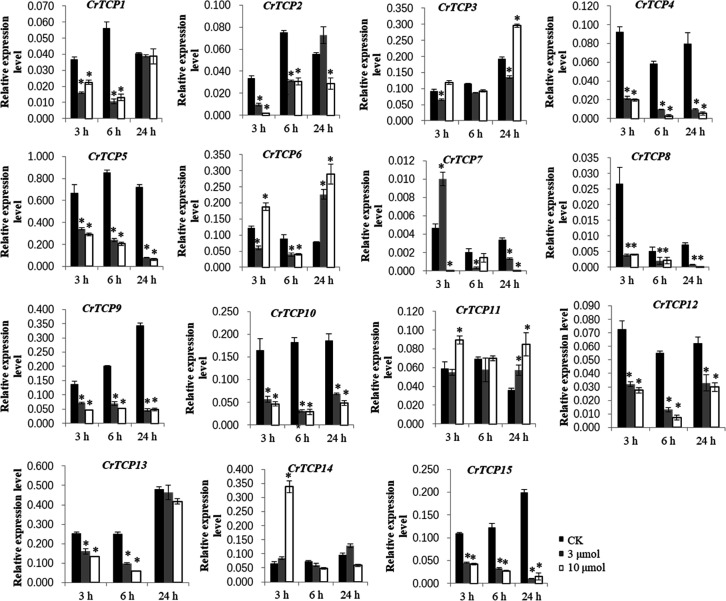
Expression analysis of CrTCP family genes under UV-B radiation. Black blocks indicated the control, grey blocks indicated 3 μmol UV-B treatment, and white blocks indicated 10 μmol UV-B treatment. The significant variations between treated sample and control (*P* < 0.05) are indicated by ‘*’. Error bars represent mean ± SD (n = 3).

Expression pattern of CrTCP family genes under different abiotic stresses, such as the UV-B radiation and MeJA treatments, were analyzed. Under the UV-B radiation, many *CrTCP* genes, such as *CrTCP2*, *CrTCP4*, *CrTCP5*, *CrTCP8*, *CrTCP9*, *CrTCP10*, *CrTCP12*, and *CrTCP13*, and *CrTCP15*, were significantly reduced at different time points. The expression of *CrTCP14* was significantly induced under 10 μmol·m^-2^·s^-1^ UV-B radiation at 3h and was significantly reduced at 24 h. The expression of *CrTCP6* was significantly down-regulated at 6 h and was up-regulated at 24 h (**Figure 4**).

**Figure 5:**
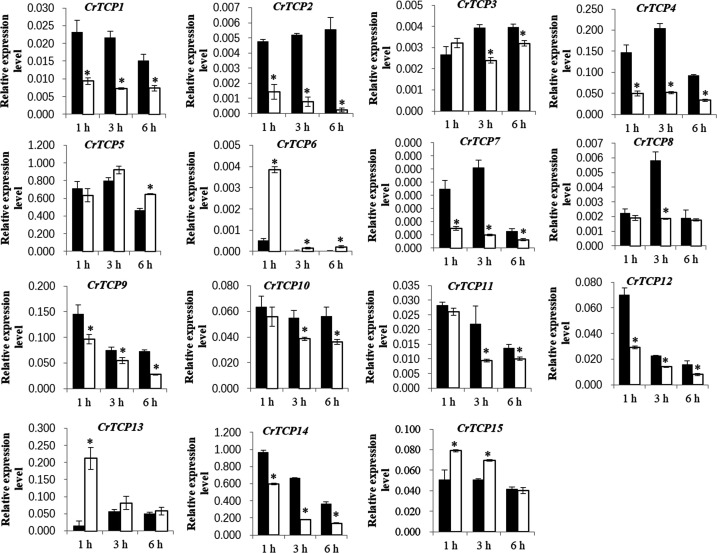
Expression analysis of CrTCP family genes under MeJA treatment. Black blocks indicated the control, and white blocks indicated 100 μmol MeJA treatment. The significant variations between treated sample and control (*P* < 0.05) are indicated by ‘*’. Error bars represent mean ± SD (n = 3).

Under the MeJA treatment, expression of *CrTCP1*, *CrTCP2*, *CrTCP4*, *CrTCP7*, and *CrTCP14* was significantly down-regulated, while expression of *CrTCP6* and *CrTCP7* was significantly up-regulated under different time points. *CrTCP8/11* were largely reduced at 3 h MeJA treatment and *CrTCP6/13* were largely induced at 1 h MeJA treatment (**Figure 5**).

Furthermore, the expression profile of CrTCP genes under MeJA and UV-B treatments was shown on a single heatmap. Clustering analysis showed that *CrTCP6*-*CrTCP7*, *CrTCP3*-*CrTCP11*, and *CrTCP5*-*CrTCP10*-*CrTCP15* have similar expression patterns. In detail, the expression levels of *CrTCP6* and *CrTCP7* were significantly up-regulated by MeJA and 24 h-UV-B treatments. The expression levels of *CrTCP5*, *CrTCP10* and *CrTCP15* were highly up-regulated by the MeJA treatment and were greatly down-regulated by the UV-B treatments (**Figure S2**).

### 3.6 The TIA contents under various elicitors

**Figure 6:**
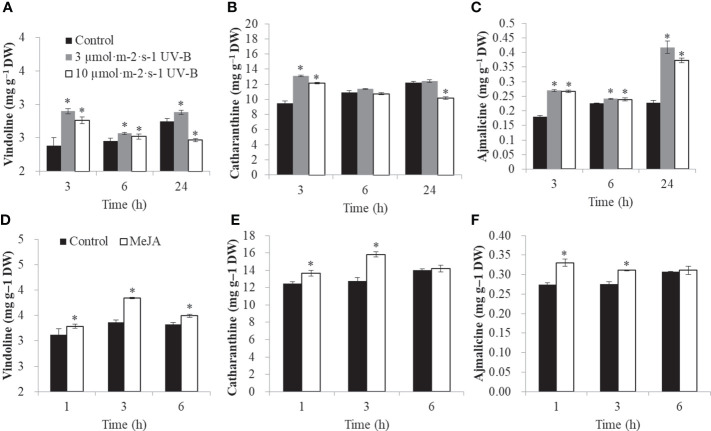
The TIA contents under various elicitors (UV-B and MeJA). UV-B radiation changed the contents of vindoline **(A)**, catharanthine **(B)**, ajmalicine **(C)**. MeJA treatment changed the contents of vindoline **(D)**, catharanthine **(E)**, ajmalicine **(F)**. The significant variations between treated sample and control (*P* < 0.05) are indicated by ‘*’. Error bars represent mean ± SD (n = 3).

To confirm the role of elicitors in TIA biosynthesis, the contents of three classic TIAs, such as vindoline, catharanthine, and ajmalicine, were determined. After 3 h, vindoline was increased from 2.39 mg.g^-1^ to 2.90 mg.g^-1^ and 2.76 mg.g^-1^ under 3 μmol·m^-2^·s^-1^ and 10 μmol·m^-2^·s^-1^ UV-B radiation, respectively. After 24 h UV-B radiation, vindoline was decreased from 2.75 mg·g^-1^ to 2.47 mg·g^-1^ under 10 μmol·m^-2^·s^-1^ UV-B radiation (**Figure 6A**). After 3 h, catharanthine was increased from 9.48 mg·g^-1^ to 13.11 mg·g^-1^ and 12.12 mg·g^-1^ under 3 μmol·m^-2^·s^-1^ and 10 μmol·m^-2^·s^-1^ UV-B radiation, respectively. After 24 h UV-B radiation, catharanthine was decreased from 12.24 mg.g^-1^ to 10.17 mg.g^-1^ under 10 μmol·m^-2^·s^-1^ UV-B treatment (**Figure 6B**). At time point 3 h, the contents of ajmalicine were induced from 0.180 mg.g^-1^ to 0.27 mg.g^-1^ and 0.27 mg.g^-1^ under 3 μmol·m^-2^·s^-1^ and 10 μmol·m^-2^·s^-1^ UV-B radiation, respectively. At time point 24 h, the contents of ajmalicine were up-regulated from 0.23 mg.g^-1^ to 0.42 mg.g^-1^ and 0.37 mg.g^-1^ under 3 μmol·m^-2^·s^-1^ and 10 μmol·m^-2^·s^-1^ UV-B treatment, respectively (**Figure 6C**).

Under MeJA treatment, vindoline was significantly up-regulated from 3.36 mg.g^-1^ to 3.85 mg·g^-1^ at 3 h (**Figure 6D**). Catharanthine was significantly up-regulated from 12.48 mg·g^-1^ to 13.67 mg.g^-1^ and 12.78 mg·g^-1^ to 15.83 mg·g^-1^ at 1 h and 3 h, respectively (**Figure 6E**). Ajmalicine was significantly induced from 0.27 mg·g^-1^ to 0.33 mg·g^-1^ and 0.28 mg·g^-1^ to 0.31 mg·g^-1^ at 1 h and 3 h, respectively (**Figure 6F**).

### 3.7 The expression levels of several TIA biosynthesis-related genes under various elicitors

Expression pattern of several TIA pathway genes under different abiotic stresses, such as the UV-B radiation and MeJA treatments, were analyzed. Our data showed that *CrASα*, *CrTDC*, and *CrCPR* were significantly increased and *CrDXR*, *CrDXS*, *CrG10H*, *CrDAT*, and *CrPRX1* were significantly reduced under UV-B treatments (**Figure S3**). Under MeJA treatment, *CrASa*, *CrSTR*, *CrSGD*, *CrDXS*, *CrLAMT* and *CrTDC* were significantly up-regulated at different time points. Only *CrD4H* was significantly down-regulated by UV-B treatment at all three time points (**Figure S4**).

### 3.8 Prediction of potential targets of CrTCP family TFs

**Figure 7:**
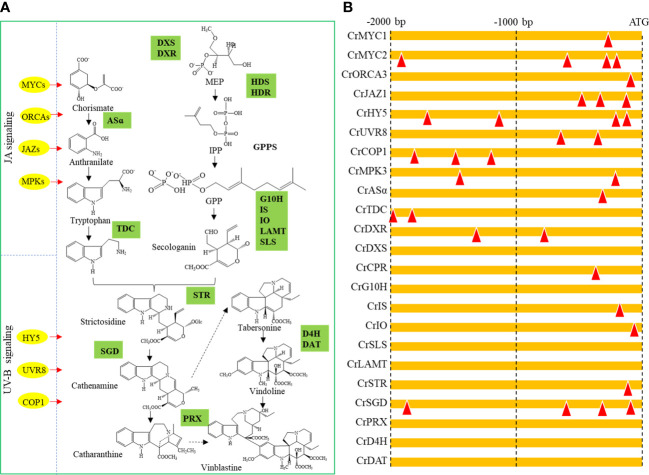
Prediction of potential targets of CrTCP family TFs. **(A)** Overview of the regulation diagram of *C. roseus* TIA biosynthesis. Yellow dots indicated upstream TFs, and green boxes indicated TIA biosynthesis-related genes. **(B)** Prediction of TCP binding element in the promoters of potential TCP downstream targets. Red triangles indicated potential TCP TF binding elements.

The consensus DNA binding sequences of TCP family TFs are two overlapping elements: GGNCCCAC and GTGGNCCC (Kosugi and Ohashi, 2002). The 2000 bp promoter regions of five JA signaling-related TF genes (*CrMYC1*, *CrMYC2*, *CrORCA3*, *CrMPK3* and *CrJAZ1*), three UV-B signaling-related TF genes (*CrHY5*, *CrUVR8* and *CrCOP1*), and ten TIA biosynthesis-related genes (*CrASα*, *CrTDC*, *CrDXR*, *CrDXS*, *CrCPR*, *CrG10H*, *CrIS*, *CrIO*, *CrSLS*, *CrLAMT*, *CrSTR*, *CrSGD*, *CrPRX*, *CrD4H* and *CrDAT*) were scanned for the TCP binding elements (TBEs) (**Figure 7A**). All selected TF promoters contain at least one TBS. The promoters of *CrMYC2* and *CrHY5* contain four TBEs. For the TIA biosynthesis-related genes, the promoters of *CrASα*, *CrDXR*, *CrCPR*, *CrIS*, *CrIO*, *CrSTR* and *CrSGD* contain at least one TBE (**Figure 7B**).

## 4 Discussion

*C. roseus* is a natural medicinal plant that has been used since ancient times (Kumar et al., 2022). Different fractions of *C. roseus* extract have been proved to have various pharmacological effects, such as anti-microbial, anti-oxidant, and anti-cancer (Das et al., 2020). Due to the extremely low contents, active ingredients from *C. roseus* are expressive and have great demand in pharmaceutical market (Yamamoto et al., 2016). The significance of *C. roseus* in a modern medical system makes the genetic breeding being an important research field.

TCP TFs are a common family with redundant functions in plants (Zhou et al., 2022). With release of the scaffolded genome of *C. roseus*, it is necessary to annotate the TCP genes at the whole-genome level. Recently, an improved *C. roseus* genome assembly composed of 2090 scaffolds with an N50 scaffold size of 2,579,454 bp was published, giving the possibility of further functional researches (Franke et al., 2019). Based on the newly released *C. roseus* genome, 15 TCP family members were identified, which is less than numbers of TCP family members in model plants (Navaud et al., 2007; Li, 2015). Limited to the quality of genome sequencing and assembly, not all TCP members have been identified. Once the high-quality genome at the chromosome level is available, deep analysis of TCP family can be further carried out.

Analysis of gene evolution relationship is helpful to predict gene function. AtTCP11, a class I TCP, is a developmental regulator involved in leaf, stem, petiole, and pollen development (Viola et al., 2011). CrTCP3, a homologous protein of AtTCP11, might be also a multifunctional growth regulator. In *Arabidopsis*, CIN-like AtTCP13 is another plant growth regulator under dehydration stresses (Urano et al., 2022). CrTCP13 is a close homologous protein of AtTCP13, indicating potential function of CrTCP13 in stress responses. Recently, AtTCP20 was reported to be involved in maintaining auxin homeostasis by modulating the expression of *GH3.3* gene (Kong et al., 2022). CrTCP11 showed a close relationship to AtTCP13, indicating that CrTCP13 might be a regulator in hormone signaling pathways.

A large number of active ingredients are enriched in the leaves and flowers of *C. roseus*. The methanolic extracts of *C. roseus* leaves have incredible contributions to the field of medicinal applications. (Rajashekara et al., 2022). *C. roseus* flower extracts have a significant antifungal property and are used as antibacterial agents to combat different infections (Neglo et al., 2022). Tissue-specific expression analysis revealed that *CrTCP2*, *CrTCP4*, and *CrTCP7* genes might be involved in leaf-specific accumulation of active ingredients and *CrTCP8*, *CrTCP10*, and *CrTCP13* genes might be involved in flower-specific accumulation of active ingredients.

TFs regulated gene expression at the transcriptional level by binding to different *cis*-acting elements (Yu et al., 2022a; Zhan et al., 2022; Feng et al., 2023). A series of regulatory elements have been identified in the promoter region of *TCP* genes, including growth-related elements, stress-responsive elements, and hormone signaling-related elements (Feng et al., 2018). Due to the presence of different types of stress *cis*-elements in promoter regions, *CrTCP12*, *CrTCP8*, *CrTCP7*, *CrTCP3*, *CrTCP10*, and *CrTCP11* might be associated with multiple stress responses. Several active ingredients play important roles in the adaptation of plants to the adverse environments, and some environmental stresses can be used as elicitors (Ramakrishna and Ravishankar, 2011; Hao et al., 2022). In this study, the positive roles of UV-B radiation and MeJA application in the induction of TIA contents were confirmed. However, the roles of TCP members in UV-B- and MeJA-mediated TIA biosynthesis are largely unknown. In *Ginkgo biloba*, the expression of *GbTCP06*, *GbTCP11*, and *GbTCP13* was significantly up-regulated by exogenous of MeJA application (Yu et al., 2022b). In Chinese flowering cabbage, BrTCP7 is associated with MeJA-mediated leaf senescence by activating JA signaling pathway and chlorophyll metabolism (Xu et al., 2019). In *C. roseus*, several UV-B-inhibited CrTCPs, such as *CrTCP4*, *CrTCP5*, *CrTCP8*, *CrTCP9*, *CrTCP10*, *CrTCP12*, and *CrTCP15*, and MeJA-inhibited CrTCPs, such as *CrTCP1*, *CrTCP2*, *CrTCP4*, *CrTCP7*, and *CrTCP14*, were identified by qRT-PCR assay, suggesting that they might be negative regulators in the stress-induced biosynthesis of TIAs.

In *C. roseus*, a series of JA-responsive TF genes, such as *CrMYC2* and *ORCA3*, were reportedly involved in the TIA biosynthesis (Paul et al., 2017). Previous studies revealed that the TCP proteins tend to form a homodimer or a heterodimer, and two variable and overlapping consensus sequences, GGNCCCAC and GTGGNCCC, were identified to be TBEs (Kosugi and Ohashi, 2002). In our study, the promoters of *CrMYC2* and *CrORCA3* contained four and one TBEs, respectively, suggesting that they were potential downstream targets of JA-responsive TCPs. COP1, HY5, and UVR8 are important regulators in UV-B signaling pathway (Zhang et al., 2022). The promoters of *CrUVR8* and *CrCOP1* contained two and three TBEs, respectively, suggesting that they were potential downstream targets of UV-B-responsive TCPs. Additionally, promoter analysis also showed that CrTCPs might be involved in TIA biosynthesis by directly binding to the promoters of *CrASα*, *CrTDC*, *CrDXR*, *CrCPR*, *CrIS*, *CrIO*, *CrSTR*, and *CrSGD*. To confirmed the roles of CrTCPs in the regulation of TIA pathway, the expression levels of several key TIA pathway genes were determined using qRT-PCR assay. As part of the iridoid pathway, CPR catalyzes the conversion of geraniol to 10-hydroxygeraniol (Miettinen et al., 2014). Tryptamine, an important precursor of TIS biosynthesis, is catalyzed from tryptophan by TDC (Goddijn et al., 1994). In our study, the expression of *TDC* and *CPR* genes was significantly up-regulated by the UV-B treatment. Considering that *TDC* and *CPR* are potential targets of TCP TFs, it showed that TCPs might plat important roles in UV-B-mediated TIA accumulation in *C. roseus*. The joining of secologanin and tryptamine is the key step to synthesize strictosidine, which is catalyzed by STR (Li and Gibson, 2021). Finally, SGD hydrolyses the strictosidine glucose to form a dialdehyde for Cathenamine biosynthesis (van Der Heijden et al., 2004). Under MeJA treatment, the expression of *CrSTR* and *CrSGD* were increased. Interestingly, the promoter of CrSTR contained one TBE, and the promoter of CrSGD contained four TBEs, suggesting that TCPs played a potential role in MeJA-induced TIA biosynthesis.

## 5 Conclusion

In the present study, a total of 15 CrTCPs were identified from in the genome of *C. roseus*. All CrTCP proteins were divided into three subgroups, P-type, C-type, and CYC/TB1, according to their conserved TCP domain. Analyses of gene structures, conserved motifs, and *cis*-elements was performed to get insights into *TCP* genes in *C. roseus*. The expression profiling of CrTCP family members was appraised in different tissues and under two elicitors. Three *CrTCP* genes, including *CrTCP2*, *CrTCP4*, and *CrTCP7*, were highly expressed in the leaves, and four *CrTCP* genes, including *CrTCP13*, *CrTCP8*, *CrTCP6*, and *CrTCP10*, were greatly expressed in the flowers. Several MeJA- and UV-B- responsive *TCP* genes were identified. Promoter analysis showed that MeJA responsive TFs (*CrMYC2* and *CrORCA3*), UV-B radiation responsive TFs (*CrUVR8* and *CrCOP1*), and TIA biosynthesis-related genes (*CrASα*, *CrTDC*, *CrDXR*, *CrCPR*, *CrIS*, *CrIO*, *CrSTR*, and *CrSGD*) might be potential downstream target of CrTCPs. Furthermore, the expression patterns of TIA biosynthesis-related genes under UV-B and MeJA treatments were obtained. Overall, our results provided actual data and theoretical basis for the functional identification of TCPs in *C. roseus*.

## Data availability statement

The original contributions presented in the study are included in the article/**Supplementary Material**. Further inquiries can be directed to the corresponding authors.

## Author contributions

JH, HQZ and CS conceptualized the initial study. CS, HQZ, JH, LZ, YH, HSZ, HK, X.L., ZTW and CC were involved in the experimental layout. JH, HK, CC, JQ, ZL and ZJW performed the lab experiments, HQZ and CS drafted the initial article. All authors contributed to the article and approved the submitted version.
