# Supplementary material for: Genome-wide identification and expression analysis of TCP family genes in Catharanthus roseus
Source: Front Plant Sci. 2023 Apr 12;14:1161534. doi: 10.3389/fpls.2023.1161534 (PMC10130365; doi:10.3389/fpls.2023.1161534)
Supplement: Supplementary file 1 [file DataSheet_1.pdf]

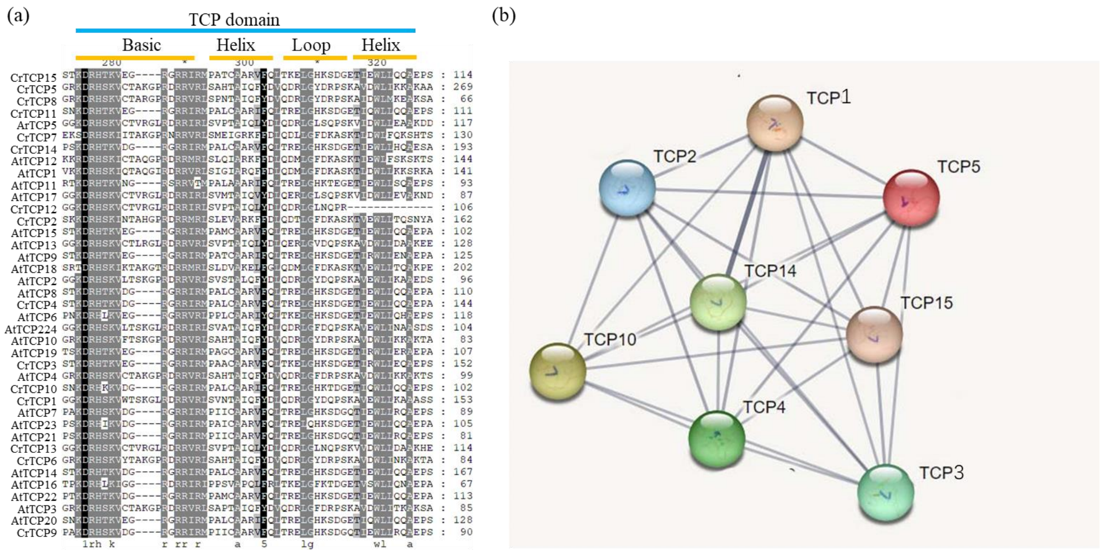

Figure S1 Analysis of CrTCP family members. (a) The alignment analysis of CrTCP family members. (b) The protein-protein network between all CrTCPs was predicted using STRING tool.
