# Supplementary material for: Genome-wide identification and expression analysis of TCP family genes in Catharanthus roseus
Source: Front Plant Sci. 2023 Apr 12;14:1161534. doi: 10.3389/fpls.2023.1161534 (PMC10130365; doi:10.3389/fpls.2023.1161534)
Supplement: Supplementary file 2 [file DataSheet_2.pdf]

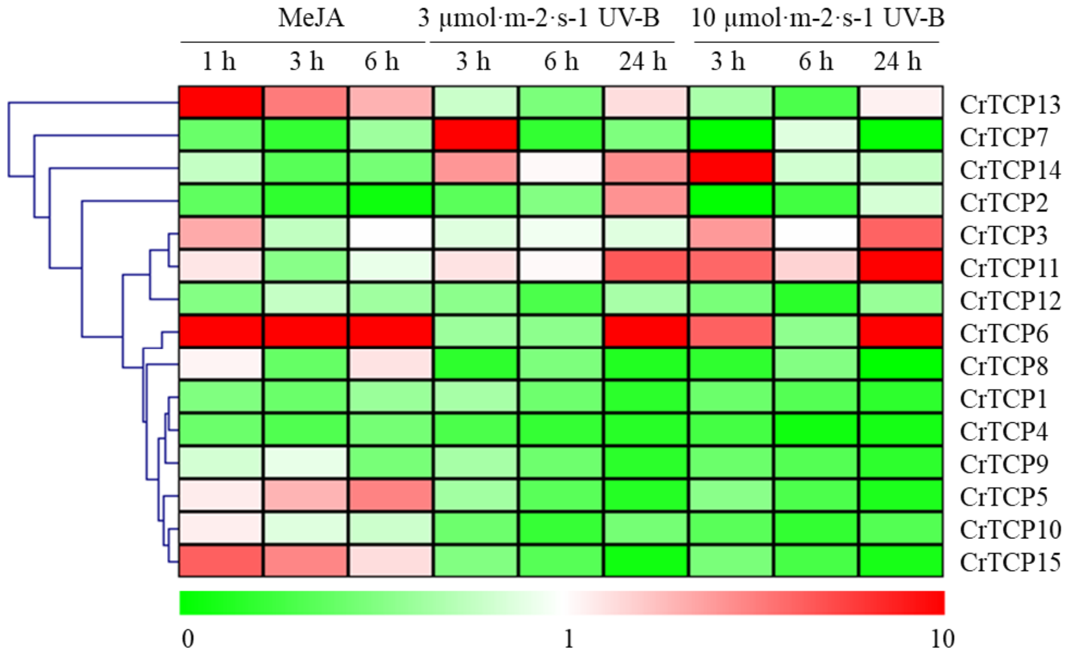

Figure S2 **The expression profile of CrTCP genes under MeJA and UV-B treatments.** The heatmap scale ranges from – 1.5 to +1.5 on a log2 scale.
