# Supplementary material for: Genome-wide identification and expression analysis of TCP family genes in Catharanthus roseus
Source: Front Plant Sci. 2023 Apr 12;14:1161534. doi: 10.3389/fpls.2023.1161534 (PMC10130365; doi:10.3389/fpls.2023.1161534)
Supplement: Supplementary file 3 [file DataSheet_3.pdf]

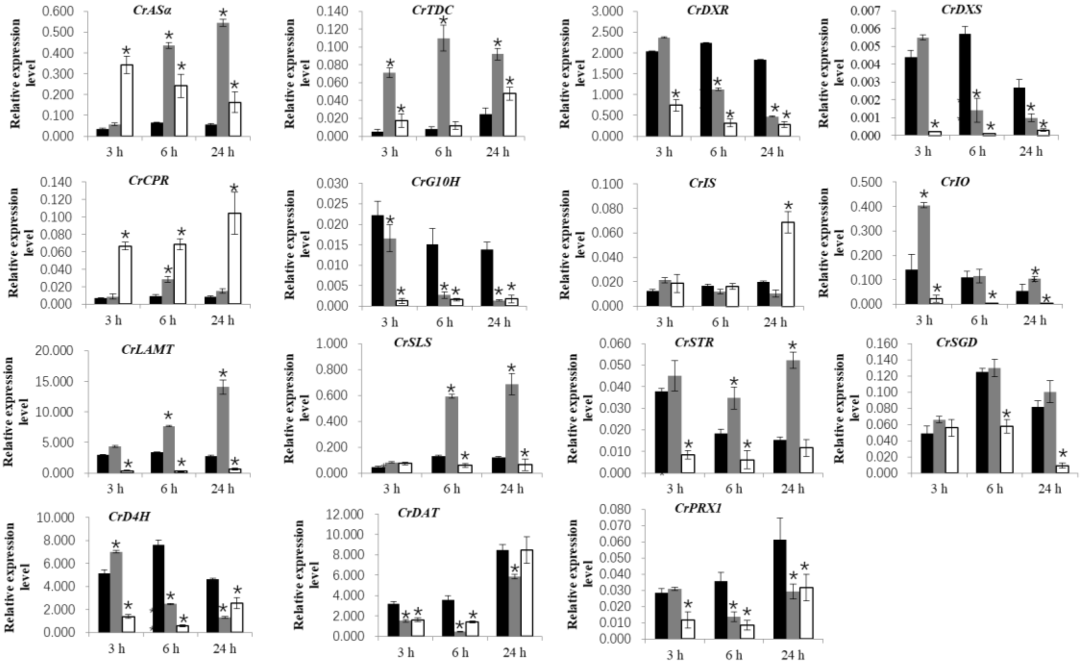

Figure S3 The expression levels of several TIA biosynthesis-related genes under UV-B treatments. The significant variations between treated sample and control ($P < 0.05$) are indicated by ‘*’. Error bars represent mean ± SD (n = 3).
