# Supplementary material for: Genome-wide identification and expression analysis of TCP family genes in Catharanthus roseus
Source: Front Plant Sci. 2023 Apr 12;14:1161534. doi: 10.3389/fpls.2023.1161534 (PMC10130365; doi:10.3389/fpls.2023.1161534)
Supplement: Supplementary file 5 [file Table_1.doc]

**Supplemental file 1** The HPLC chromatograms for measurement of terpenoid indole alkaloid contents

Standards of vindoline, catharanthine , ajmalicine

Control, 1h（repeat 1）

Control, 1h（repeat 2）

Control, 1h（repeat 3）

Control, 3h（repeat 1）

Control, 3h（repeat 2）

Control, 3h（repeat 3）

Control, 6h（repeat 1）

Control, 6h（repeat 2）

Control, 6h（repeat 3）

MeJA, 1h（repeat 1）

MeJA, 1h（repeat 2）

MeJA, 1h（repeat 3）

MeJA, 3h（repeat 1）

MeJA, 3h（repeat 2）

MeJA, 3h（repeat 3）

MeJA, 6h（repeat 1）

MeJA, 6h（repeat 2）

MeJA, 6h（repeat 3）

Control, 3h（repeat 1）

Control, 3h（repeat 2）

Control, 3h（repeat 3）

Control, 6h（repeat 1）

Control, 6h（repeat 2）

Control, 6h（repeat 3）

Control, 24h（repeat 1）

Control, 24h（repeat 2）

Control, 24h（repeat 3）

3 µmol·m-2·s-1 UV-B, 3h（repeat 1）

3 µmol·m-2·s-1 UV-B, 3h（repeat 2）

3 µmol·m-2·s-1 UV-B, 3h（repeat 3）

3 µmol·m-2·s-1 UV-B, 6h（repeat 1）

3 µmol·m-2·s-1 UV-B, 6h（repeat 2）

3 µmol·m-2·s-1 UV-B, 6h（repeat 3）

3 µmol·m-2·s-1 UV-B, 24h（repeat 1）

3 µmol·m-2·s-1 UV-B, 24h（repeat 2）

3 µmol·m-2·s-1 UV-B, 24h（repeat 3）

10 µmol·m-2·s-1 UV-B, 3h（repeat 1）

10 µmol·m-2·s-1 UV-B, 3h（repeat 2）

10 µmol·m-2·s-1 UV-B, 3h（repeat 3）

10 µmol·m-2·s-1 UV-B, 6h（repeat 1）

10 µmol·m-2·s-1 UV-B, 6h（repeat 2）

10 µmol·m-2·s-1 UV-B, 6h（repeat 3）

10 µmol·m-2·s-1 UV-B, 24h（repeat 1）

10 µmol·m-2·s-1 UV-B, 24h（repeat 2）

10 µmol·m-2·s-1 UV-B, 24h（repeat 3）
